# TALOs, Fill the Gap: Tafasitamab and Lenalidomide in Diffuse Large B‐Cell Lymphoma in the Real‐Life Patient Journey

**DOI:** 10.1002/hon.70167

**Published:** 2026-01-14

**Authors:** Lisa Argnani, Cinzia Pellegrini, Ombretta Annibali, Piera Angelillo, Enrico Amaducci, Filippo Ballerini, Flaminia Bellisario, Andrea Bernardelli, Riccardo Bruna, Catello Califano, Giusy Cetani, Giulia Daghia, Enrico Derenzini, Antonio Frolli, Francesco Gaudio, Valerio Guarente, Ausilia Gorgone, Liardo Eliana Valentina, Elisa Lucchini, Dario Marino, Luca Nassi, Paolo Nicoli, Mattia Novo, Francesca Palombi, Caterina Patti, Vincenzo Pavone, Marcello Riva, Filomena Russo, Greta Scapinello, Alessandro Severino, Monica Tani, Daniele Vallisa, Beatrice Casadei, Alessandro Broccoli, Pier Luigi Zinzani

**Affiliations:** ^1^ Dipartimento di Scienze Mediche e Chirurgiche (DIMEC) Alma Mater Studiorum Università of Bologna Bologna Italy; ^2^ IRCCS IRCCS Azienda Ospedaliero‐Universitaria di Bologna Istituto di Ematologia “Seràgnoli” Bologna Italy; ^3^ UOS DH Ematologico Area Ematologia Trapianto Cellule Staminali Fondazione Policlinico Universitario Campus Bio‐Medico Roma Italy; ^4^ Lymphoma Unit Hematology and Bone Marrow Transplantation Unit Department of OncoHematology IRCCS San Raffaele Scientific Institute Milano Italy; ^5^ A.O. Citta della Salute e della Scienza Torino Italy; ^6^ Ospedale Policlinico San Martino—IRCCS Genova Italy; ^7^ Fondazione Policlinico Universitario Agostino Gemelli IRCCS Roma Italy; ^8^ Hematology Unit Azienda Ospedaliera Universitaria Integrata Verona Italy; ^9^ Division of Hematology Department of Translational Medicine Università del Piemonte Orientale and Azienda Ospedaliero‐Universitaria Maggiore della Carità Novara Italy; ^10^ ASL Salerno Salerno Italy; ^11^ AO Sant'Anna e San Sebastiano di Caserta Caserta Italy; ^12^ U.O. di Ematologia ‐ Dipartimento di Oncologia e Medicine Specialistiche Azienda Ospedaliero Universitaria di Ferrara Cona Ferrara Italy; ^13^ Clinical Haemato‐Oncology Division IEO European Institute of Oncology Milano Italy; ^14^ SCDU Ematologia AO Mauriziano Torino Italy; ^15^ Hematology and Stem Cell Transplantation Unit AOU Consorziale Policlinico Bari Italy; ^16^ Department of Medicine and Surgery LUM University Bari Italy; ^17^ UOC Oncoematologia Ospedale “S. Bassiano” di Bassano del Grappa AULSS7 Pedemontana Vicenza Italy; ^18^ UOC Ematologia ARNAS Garibaldi Catania Italy; ^19^ IRCCS Istituto Romagnolo per lo Studio dei Tumori (IRST) “Dino Amadori” Meldola Italy; ^20^ UOC Ematologia Azienda Sanitaria Universitaria Giuliano Isontina Ospedale Maggiore Trieste Italy; ^21^ Istituto Oncologico Veneto IRCCS Padova Italy; ^22^ Hematology Careggi Hospital and University of Florence Florence Italy; ^23^ Azienda Ospedaliera Universitaria San Luigi Gonzaga Orbassano Italy; ^24^ Hematology Division ‐ A.O.U. Città della Salute e della Scienza di Torino Turin Italy; ^25^ Istituto Nazionale Tumori Regina Elena IRCCS I.F.O. Roma Italy; ^26^ Oncohematology Unit A.O.O.R. Villa Sofia Cervello Palermo Italy; ^27^ A.O.Card.G.Panico U.O.Ematologia e TMO Tricase Italy; ^28^ Hematology Unit San Bortolo Hospital AULSS 8 Berica Vicenza Italy; ^29^ Ematologia e CTMO Ospedale Maggiore di Parma Parma Italy; ^30^ Azienda Ospedale‐Università di Padova Padova Italy; ^31^ AOU Federico II Napoli Italy; ^32^ UOC Ematologia Ospedale S.Maria delle Croci Ravenna Italy; ^33^ UO Ematologia e CTMO Ospedale G da Saliceto Piacenza Italy

**Keywords:** diffuse large B‐cell lymphoma, lenalidomide, real‐life, tafasitamab

## Abstract

The combination of tafasitamab and lenalidomide (tafa‐lena) has demonstrated efficacy in relapsed/refractory diffuse large B‐cell lymphoma (R/R DLBCL), as evidenced by the L‐MIND study. To investigate the therapeutic potential and safety profile of tafa‐lena in real‐life, we conducted a national multicentric retrospective study. Eighty‐three patients with median age of 74 years were enrolled. The 49.4% of patients had a disease refractory to the first line therapy while 63.9% were refractory to the most recent one. The best overall response rate was 47% (28.9% complete remission). With a median follow‐up of 16 months, the median overall survival (OS) was 8.6 months and the median progression‐free survival (PFS) was 4.5 months. Disease‐free survival and median duration of response were reached at 52.8 and at 52.1 months, respectively. Compared to refractory disease (*N* = 53, 63.9%), relapsed disease (*N* = 26, 31.3%) was associated with better outcome in term of PFS (median 2.8 vs. 12.4 months) and OS (median 5.4 months vs. not reached). Neutropenia (52.5%) was the most common adverse events, predominantly related to lenalidomide. Our findings align with other real‐world studies, supporting the regimen as effective and safe, and highlighting that one third of patients experiencing long‐lasting responses even with dose reduction.

## 1 Introduction

For years, first‐line therapy of diffuse large B‐cell lymphoma (DLBCL) was based on the combination of rituximab, cyclophosphamide, doxorubicin, vincristine, and prednisone (R‐CHOP) and eligibility for high‐dose chemotherapy (HDCT) and autologous stem cell transplantation (ASCT) after response to salvage chemoimmunotherapy in relapsed/refractory (R/R) patients [1, 2]. Recent advances in the molecular characterization of this disease resulted in the development and approval of many new drugs and treatment regimens, especially in the R/R setting. As a result, Hematologists have many active agents to choose from. However, the main problem is that a comprehensive treatment algorithm for these agents, which we could classify as competitive, has not yet been shared [3]. This is partly due to the lack of comparative studies and in partly to the scarcity of real‐life reports. In particular, the latter often do not report results in a uniform manner or with the same variables as registration studies, causing bias in the interpretation of efficacy and safety data [4, 5, 6]. Among novel agents for R/R DLBCL are included: CAR T‐cells (CAR‐T) therapy, polatuzumab vedotin, loncastuximab tesirine, tafasitamab plus lenalidomide (tafa‐lena), bispecific antibodies, and the “younger” brentuximab vedotin associated with lenalidomide and glofitamab plus gemcitabine and oxaliplatin [7, 8]. In addition, there are many ongoing clinical trials with new agents/combination [9].

Even if CAR‐T technology is currently the more promising in both second‐ and third‐line setting, its large‐scale use is not yet possible due to several factors: from the absence of effective bridging therapies to the severe toxicities that often occur, to logistical problems such as high costs and the need for specialized Centers.

In this scenario, tafasitamab, a humanized monoclonal antibody (mAb) directed against the pan B‐cell antigen CD19 and that is produced by recombinant DNA technology in Chinese hamster ovary cells has been investigated in combination with other therapeutic agents such as the immunomodulatory drug lenalidomide that modulates pro‐inflammatory cytokines and T‐cell co‐stimulation [10].

L‐MIND is a phase II, single‐arm, open‐label, multicenter study which investigated the safety and efficacy of lenalidomide combined with tafasitamab in patients with R/R DLBCL who were not eligible for an HDCT followed by ASCT. The study reported a 57.5% of objective response rate with 43% of complete response (CR) with a manageable toxicity that led to the approval by regulatory agencies [11, 12, 13].

At the time of writing, only few real‐world full papers and congress abstracts were published [14, 15, 16, 17, 18, 19, 20, 21]. Thus, following the EMA approval [21] and the granting of early access to tafasitamab plus lenalidomide to Italian eligible patients through a Named Patient Program (NPP), we conceived to collect all data to assess the actual effectiveness and safety of this new regimen.

## 2 Subjects and Methods

Data from patients treated with tafa‐lena outside a controlled clinical trial could give additional information about the clinical use, treatment duration, effectiveness, and safety profile of this regimen in a real‐life context before widespread diffusion after formal regulatory approval. TALOs is a retrospective multicenter study that aimed to retrospectively evaluate the effectiveness and tolerability of tafa‐lena in patients with R/R DLBCL treated with at least one dose of tafasitamab in association with lenalidomide under the NPP, (D.M. 7 Sep 2017), in the period from April 2022 to December 2022.

The start of patient recruitment/selection was subjected to and followed the approval of the study by the Ethics Committee of the Coordinating Center (approval id 442/2023/Oss/AOUBo). This study adhered to the standards of the Helsinki Declaration. All patients were enrolled consecutively after providing written informed consent or authorization from the privacy guarantor for patients who were lost to follow‐up or died.

No formal sample size estimation was made as, after a national feasibility, all the Center who have adhered to the NPP were involved in the present study and all the patients treated were enrolled obtaining a census of 83 patients. The primary study endpoint was the overall response rate (ORR, the sum of complete response [CR] and partial response [PR] rate) intended as both final responses achieved and best responses reached at any timepoint during treatment. Secondary endpoints were Duration of Response (DoR), Progression‐free survival (PFS), Overall survival (OS), Time‐to‐Next‐Treatment (TTNT) and type, incidence, severity of any adverse events (AE) occurred from start of treatment to 30 days after last infusion and their possible relationship with study drugs.

Initially, descriptive analyses were conducted to evaluate the demographic and clinical characteristics of the patient cohort at baseline. AEs and serious AEs (SAE) were tabulated in detail and their incidence was calculated; the severity of AEs was defined in accordance with the CTCAE v. 5.0 (Common Terminology Criteria for AEs). To evaluate the associations between the variables of interest, association tests were used, such as the Chi‐square test and Fisher's exact test, depending on the distributions of the variables and the numerousness. For quantitative variables, the classic Student's t‐test and the non‐parametric Wilcoxon‐Mann‐Whitney Test, also known as Mann‐Whitney *U* test, were employed.

Time‐to‐point events were estimated using the Kaplan‐Meier method. Comparison of survival across subgroups were performed using Kaplan–Meier product‐limit survival curve estimates and log‐rank tests or the Wilcoxon‐Breslow‐Gehan test, as applicable. The Cox regression model was used to evaluate the effect of the predictor factors on patient outcomes, controlling for any confounding variables. Univariate and multivariate analyses were conducted and hazard ratio (HR) was reported. Subsequent to the estimation of the models, a backward stepwise regression was conducted for the latter (Supplementary Results, Tables S1 and S2). It is a stepwise regression approach that, starting from a saturated model, removes the variable with the highest *p* at each step, until all remaining variables have a *p* value within the established threshold (< 0.05). The reduced model obtained is the one that best explains the variability of the data and is useful because the number of predictors is reduced, decreasing the problem of multicollinearity. Median follow‐up was estimated with the reverse Kaplan‐Meier method.

All analyses were conducted using STATA 18/SE statistical software (StataCorp LP, TX) and results were considered significant for *p* less than 0.05.

## 3 Results

### 3.1 Patients Characteristics

Eighty‐three patients were enrolled at 31 Centers covering almost all geographic areas in Italy. The characteristics of the 83 patients with R/R DLBCL who received at least one dose of tafa‐lena are summarized in Table 1. Fifty‐two patients were male (62.7%) and 31 female (37.3%). The median age at diagnosis was 70 years (range 33–88) and the median age at initiation of therapy was 74 years (range 39–91). Seven patients (8.4%) had double‐hit disease. At baseline, 76 patients (91.6%) had measurable disease, 22 patients (26.5%) had B symptoms, 20 patients (24.1%) presented with a bulky mass and 46 patients (55.4%) with extranodal involvement.

**TABLE 1:** Patients characteristics at baseline.

Characteristics (*N* = 83)	
Male, *n* (%)	52 (62.7)
Female, *n* (%)	31 (37.3)
Age at baseline, years (range), IQR	74 (39–91), 14
Age at diagnosis, years (range), IQR	70 (33–88), 13
Double‐hit, *n* (%)	7 (8.4)
Outcome first line, *n* (%)	
Refractory	40 (48.2)
Relapsed	41 (49.4)
NA	2 (2.4)
Previous ASCT, *n* (%)	6 (7.2)
Ann Arbor stage at diagnosis, *n* (%)	
1	1 (1.2)
2	11 (13.3)
3	26 (31.3)
4	43 (51.8)
NA	2 (2.4)
Measurable disease, *n* (%)	76 (91.6)
Extranodal diseases, *n* (%)	46 (55.4)
B symptoms, *n* (%)	22 (26.5)
ECOG, *n* (%)	
0	22 (26.5)
1	37 (44.6)
2	16 (19.3)
3	3 (3.6)
4	0 (0)
5	0 (0)
NA	5 (6)
Bulky disease, *n* (%)	20 (24.1)
LDH increased, *n* (%)	37 (44.6)
Response to the last previous therapy, *n* (%)	
CR	25 (30.1)
PR	30 (36.2)
SD	6 (7.2)
PD	18 (21.7)
NA	4 (4.8)
Outcome to the last previous therapy, *n* (%)	
Refractory	53 (63.9)
Relapsed	26 (31.3)
NA	4 (4.8)
Median time from to the last previous therapy to tafa‐lena, months (range)	1.7 (0–27.1)

Abbreviations: ASCT: autologous stem cell transplantation; CR: complete response; IQR: interquartile range; NA: not available; PD: progression of disease; PR: partial response; SD: stable disease.

Regarding the Ann Arbor stage, 12 patients (14.5%) had limited‐stage (1 patient in stage I and 11 patients in stage II) while 69 patients (83.1%) had advanced‐stage disease (26 patients in stage III and 43 patients in stage IV). On laboratory tests, 37 patients (44.6%) had increased LDH values, in most cases grade 1 (26 patients) or 2 (7 patients).

Regarding the outcome of first‐line therapy, 40 patients (48.2%, in detail 34/40 after [mini]R‐CHOP or R‐CHOP like regimens) were refractory, and 41 patients (49.4%) relapsed after initial response. At the time of inclusion in the study, patients had received a median of 2 therapies (range 1–7). Most cases (38.6%) had previously undergone only one line of therapy.

Different treatment schedules were administered as last therapy before tafa‐lena. The most common were R‐CHOP/R‐COMP (15 patients plus 8 mini‐R‐CHOP/R‐COMP) and R‐pola‐bendamustine (6 patients). In particular all but one of the 6 patients who had received R‐pola‐bendamustine had progression of disease as best response. The sixth patient achieved a CR but resulted as refractory as relapsed after only 5 months. Regardless of the type of therapeutic regimen used, 25 patients (30.1%) achieved a CR, 30 patients (36.2%) achieved a PR, 6 patients (7.2%) achieved SD and 18 patients (21.7%) a PD as a response to the last previous therapy. Overall, 53 patients (63.9%) were refractory to the last prior therapy and 26 patients (31.3%) had relapsed, with a median time from the last therapy to the combination of tafa‐lena of 1.7 months (range 0.5–27.1).

### 3.2 Treatment

Patients received tafasitamab infusion for a number of cycles between 1 and 19 (median = 3, range 1–19). In 37 patients (44.6%) the dosage was reduced due to toxicity.

Patients received lenalidomide orally for 1 to 16 cycles (median = 2, range 1–16) with a median lenalidomide dosage of 25 mg (range 10–25). A median of 3 cycles (range 1–18) were performed at a reduced dosage and 38 patients (45.8%) discontinued lenalidomide during therapy, of which 13 temporarily and 22 permanently. Twenty‐three patients (27.7%) received tafasitamab monotherapy, with a median of 2 cycles (range 1–12).

### 3.3 Effectiveness and Outcomes

At the end of therapy, CR was achieved by 17 patients (20.5%) and PR by 10 patients (12.1%); 5 patients (6%) resulted in stable disease (SD) and 50 patients (60.2%) had progression of disease (PD). The ORR was 32.6%, with 27 of 83 patients achieving a complete or partial response to treatment.

As best response to therapy, obtained at any time of treatment, 24 patients (28.9%) obtained a CR and 15 patients (18.1%) a PR, leading to an ORR of 47%, while 34 patients (41%) had a PD.

**FIGURE 1:**
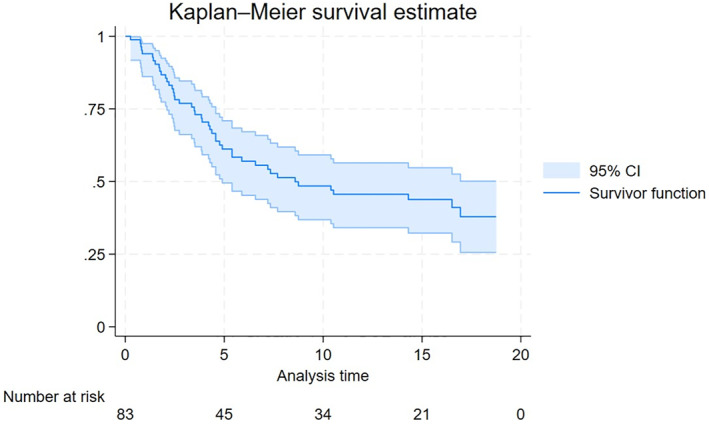
Overall survival.

**FIGURE 2:**
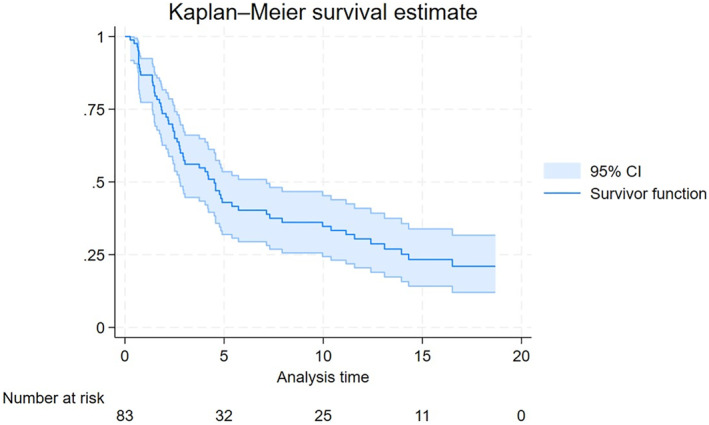
Progression‐free survival.

With a median follow up of 16 months, the median OS was 8.59 months (Figure 1). The median PFS was 4.5 months (Figure 2). For the 24 patients who achieved a CR as best response the estimated median DFS was 52.8 months (68.2% at 12 months), while for those patients obtaining at least a partial response (39 patients) the median DoR was 51.2 months.

After tafa‐lena, 38 patients (45.8%) had disease relapse and 27 of these (32.5%) underwent subsequent therapy with a median TTNT of 3.2 months. CR at subsequent therapy was achieved in 8 patients (29.6%) and PR in 2 patients (7.4%); 1 patient (3.7%) achieved SD and 10 patients (37%) achieved PD. Only one patient underwent CAR T‐cell therapy as subsequent treatment, achieving CR.

A significant association emerged between the final response and variables as Bulky Disease (*p* = 0.014 in favor of absence of this characteristic), Outcome of the last therapy prior to tafa‐lena (*p* = 0.024 in favor of relapsed patients vs. refractory), and dose reduction (*p* = 0.018, in favor to patients who did not reduce dosage of any drugs). Best response showed a significant relationship with Toxicity (*p* = 0.028), Dose Reduction (*p* = 0.021) and Outcome of the last therapy prior to tafa‐lena (*p* = 0008).

Considering the univariate model for OS (Table 2), the significant variables were ECOG (2–3), Bulky Disease, Refractoriness to the last previous therapy, Toxicity, and Dose Reduction. Patients with Refractoriness to the previous last therapy have an HR = 2.394. To compare the OS curves, both the Log‐rank Test and the Wilcoxon‐Breslow‐Gehan Test were repeated for all variables that were significant in the univariate model. The tests carried out for the ECOG variables (2–3) (*p* = 0.027), Bulky Disease (*p* = 0.004), Refractoriness to the last therapy (*p* = 0.01), Toxicity (*p* = 0.016) and Dose Reduction (*p* = 0.002) all confirmed the significant difference between the curves of the two levels of each regressor. Among the regressors listed previously, however, only Dose Reduction maintained a significant relationship in multivariable model. An HR = 0.24 shows that patients who had a Dose Reduction have a 76% lower risk of death compared to those who did not.

**TABLE 2:** Cox regression for overall survival.

	Univariate		Multivariate Variabile Età binaria	
	HR	*p*	HR	*p*
Sex (male)	1.280	0.439	3.106	**0.045**
Age (≥ 70)				
At diagnosis	1.373	0.299	1.554	0.516
At baseline	1.323	0.421	1.046	0.955
Double‐hit (yes)	2.329	0.056	2.960	0.092
Stage (III‐IV)	1.108	0.803	3.418	0.103
Previous ASCT (yes)	1.011	0.985	0.186	0.270
Extranodal disease	0.813	0.496	0.807	0.647
Primary refractory	1.536	0.159	1.853	0.235
Sintomi B symptoms	0.959	0.903	0.492	0.191
ECOG (2–3)	1.920	**0.043**	1.965	0.223
Bulky disease	2.520	**0.005**	1.316	0.603
LDH increased	1.477	0.203	0.889	0.793
Refractoriness to last therapy	2.394	**0.020**	1.522	0.519
Toxicity	0.520	0.050	1.205	0.761
Dose reduction	0.370	**0.003**	0.244	**0.004**

*Note:* Bold values are those considered statistically significant.

Abbreviations: ASCT: autologous stem cell transplantation; ECOG PS: Eastern Cooperative Oncology Group performance status.

Regarding PFS, the variables Double‐hit, Bulky Disease, Refractoriness to the last previous therapy, Toxicity, and Dose reduction were found to be significant. In particular, with an HR = 0.396, it emerged that patients who experienced toxicity during treatment have a 60% lower risk of disease progression or death compared to those who did not experience toxicity. The Log‐rank Test and the Wilcoxon‐Breslow‐Gehan Test conducted for the variables Double‐hit (*p* = 0.023), Bulky Disease (*p* = 0.001), Refractoriness to the last therapy (*p* = 0.007), Toxicity (*p* = 0.001), and Dose reduction (*p* = 0.003) all confirmed the significant difference between the curves of the two levels of each regressor. No factor held significance in the multivariate models for PFS (Table 3).

**TABLE 3:** Cox regression for progression‐free survival.

	Univariate		Multivariate	
	HR	*p*	HR	*p*
Sex (male)	1.240	0.429	1.504	0.362
Age (≥ 70)				
At diagnosis	0.967	0.896	1.051	0.926
At baseline	0.893	0.694	1.226	0.749
Double‐hit (yes)	3.023	**0.009**	2.375	0.125
Stage (III‐IV)	1.434	0.344	2.762	0.136
Previous ASCT (yes)	1.698	0.259	2.222	0.299
Extranodal disease	1.073	0.792	0.788	0.578
Primary refractory	1.529	0.108	1.880	0.144
Sintomi B symptoms	1.695	0.058	1.478	0.429
ECOG PS (2–3)	1.684	0.076	1.727	0.221
Bulky disease	2.497	**0.002**	2.011	0.103
LDH increased	1.206	0.478	0.913	0.810
Refractoriness to last therapy	2.202	**0.009**	1.707	0.284
Toxicity	0.396	**0.002**	0.616	0.380
Dose reduction	0.460	**0.005**	0.534	0.129

*Note:* Bold values are those considered statistically significant.

Abbreviations: ASCT: autologous stem cell transplantation; ECOG PS: Eastern Cooperative Oncology Group performance status.

### 3.4 Safety (Table 4)

Hematological toxicity was observed in 41 patients for a total of 61 adverse reactions: neutropenia was the most common, recorded in 32 patients (52.5% of the total hematological toxicities) followed by thrombocytopenia, in 15 patients and anemia, in 9 patients. Most hematological events were grade 3 (27 AEs) or grade 4 (19 AEs). Overall, 53 hematological adverse reactions (86.9%) were related to study regimen, in particular 1 linked to tafasitamab, 40 to lenalidomide and 12 to both drugs. Extra‐hematological toxicity was recorded in 38 patients for a total of 49 AEs: 12 grade 1, 14 grade 2, 17 grade 3 and 5 grade 4. The most common extra‐hematological toxicities were pneumonia, diagnosed in 6 patients (12.2% of the total extra‐hematological toxicities), diarrhea and skin rash which both affected 4 patients. Some patients had a significant increase in some laboratory values: 3 patients had an increase in ALT/AST transaminases and 2 patients in gamma‐glutamyl transferase and/or alkaline.

**TABLE 4:** Toxicities.

Toxicities	
Hematological, *n* (patients)	61 (41)
Grade, *n* (%)	
1	3 (4.9)^a^
2	12 (19.7)
3	27 (44.3)
4	19 (31.1)
Description, *n* (%)	
Neutropenia	32 (52.5)
Thrombocytopenia	13 (21.3)
Anemia	9 (14.8)
Thrombocytopenia	2 (3.3)
Leukopenia	2 (3.3)
Hypercalcemia	1 (1.6)
Lymphocytopenia	1 (1.6)
Pancytopenia	1 (1.6)
Drugs relationship	
Yes	53 (86.9)
No	8 (13.1)
Type of drug for relationship	
Tafasitamab	1
Lenalidomide	40
Both	12
Extra‐hematological, *n* (patients)	49 (38)
Grade, *n* (%)	
1	12 (24.5)
2	14 (28.6)
3	17 (34.7)
4	5 (10.2)
Description, *n* (%)	
Pneumonia	6 (12.2)
Diarrhea	4 (8.2)
Skin rash	4 (8.2)
AST/ALT increase	3 (6.1)
GGT	2 (4.1)
Bronchitis	1 (2.0)
Hyporexia	1 (2.0)
Mucositis	1 (2.0)
Fever	1 (2.0)
Deep vein thrombosis	1 (2.0)
Septic shock	1 (2.0)
Vomiting	1 (2.0)
Acute myeloid leukemia	1 (2.0)
Covid‐19 infection	1 (2.0)
Erythema multiforme	1 (2.0)
Hyper‐transaminasemia (not specified)	1 (2.0)
Hyperbilirubinemia	1 (2.0)
Pulmonary thromboembolism	1 (2.0)
Renal toxicity	1 (2.0)
Gastroenteritis	1 (2.0)
Other	15 (30.6)
SAE, *n* (patients)	16^b^ (15)

^a^ percentages refer to total toxicities.

^b^ all extra‐hematological toxicities.

Abbreviation: SAE: serious adverse event.

Extra‐hematological adverse reactions resulted SAEs in 16 cases (15 patients). Overall, 45 patients (54.2%) of the 83 included in the study died, of which 29 died due to PD and 16 from other causes. Among these, the most common was COVID infection, which affected 6 patients, followed by respiratory failure in 2 patients. The other causes of death in only one patient each were: secondary acute myeloid leukemia, septic shock during therapy, dermatological toxicity resulting in Multiple Organ Failure, lung adenocarcinoma, cardiac arrest, post‐transplant lymphoproliferative disease, cerebral hemorrhage and acute myocardial infarction.

## 4 Discussion

The data relating to the study sample covered by this work, which includes 83 R/R DLBCL patients (same sample size of L‐MIND trial), are the first available on the use of tafasitamab—lenalidomide combination therapy in daily clinical practice in Italy. This treatment was administered at 31 Italian hematological departments under the NPP, before it was granted marketing authorization.

In this population of patients with R/R DLBCL not eligible for ASCT, the combination of tafasitamab and lenalidomide achieved an ORR in 32.6% of patients and a CR rate in 20.5% of patients. Significantly, for patients who achieved at least a PR, the response was durable with a median duration of 51.2 months and a median DFS (for CR patients only) of 52.8 months. Overall, for the entire study population with a median follow‐up of 16 months, the median OS was 8.6 months and the median PFS was 4.5 months. To correctly interpret these results, it is necessary to consider the characteristics of the disease and the population included in the study. DLBCL is an aggressive lymphoma that evolves rapidly and requires timely initiation of first‐line therapy, following which only approximately 60% achieve a lasting CR. Approximately 40% of patients, however, develop a relapse or result refractory to first‐line approach and according to the international SCHOLAR‐1 study [22], patients with refractory disease have a median OS of approximately 6 months. The combination of tafasitamab and lenalidomide is one of the therapeutic possibilities available from the second line for patients who are not eligible to receive ASCT due to age or comorbidities or due to lack of response to salvage chemotherapy. Furthermore, the median age of patients in the TALOs study at the start of tafasitamab was 74 years, which influences both the life expectancy of affected patients and their tolerance to the administered therapies. The results obtained in the TALOs study can be compared with those of the pivotal phase II L‐MIND study and with the results of other retrospective studies conducted in the context of daily clinical practice [11, 12, 13, 14, 15, 16, 17, 18, 19, 20]. Specifically, results from the TALOs study are lower than those of the L‐MIND pivotal study, in terms of ORR, CRR, PFS and OS, although the two studies are comparable from the point of view of sample size (81 [80 evaluable patients] L‐MIND patients and 83 TALOs patients) and the median age of the patients (72 years L‐MIND and 74 years TALOs).

First results of the L‐MIND study revealed an ORR of 60% (vs. 32.6% TALOs) and a CR of 43% (vs. 20.5% TALOs), a median PFS of 12.1 months (vs. 4.5 months TALOs) and with a median follow‐up of 13.2 months, the median OS had not yet been reached (8.6 months TALOs) [12]. These differences could be related to the restrictive selection criteria of the L‐MIND clinical trial, with low‐risk disease characteristics and patients with fewer comorbidities compared to daily clinical practice. The TALOs study, in fact, also included 7 patients with double‐hit disease, patients who had received at least 3 previous lines of therapy, patients with ECOG performance status > 2 and 37 patients with elevated LDH at diagnosis. Furthermore, the percentages of refractoriness to previous therapy are also higher in the sample of the TALOs study compared to the L‐MIND one: in the first case, 40 patients (48.2%) with primary refractory disease (progression within 6 months of the first therapy) and 53 patients (63.9%) refractory to the last therapy were included, while in the second 15 patients (18.5%) refractory to the first line and 36 patients (44.4%) refractory to the last therapy were considered. In both studies, the majority of patients had advanced Ann Arbor stage disease: 61 patients (75.3%) had stage III ‐ IV in the L‐MIND sample and 69 patients (83.1%) in the TALOs sample. The data from the final analysis of the L‐MIND study, with a follow‐up of 5 years, confirmed that tafa‐lena therapy produces long‐lasting responses, in fact, after a median follow‐up of 44 months, the median DoR was not achieved. This duration of response result was also observed in the TALOs study, where a median DoR of 51.2 months (4 years) was estimated. In addition, TALOs best response rate was 47%, that is comprised in the 95% confidence interval of the best ORR of the L‐MIND study (45.9–68.5) [11, 12, 13].

Regarding toxicity, no significant differences emerged between the L‐MIND study and the TALOs study, in particular with regard to hematological toxicity, the same rate occurred (49%) being neutropenia the most prevalent in both cases. Furthermore, the TALOs study also confirmed the finding from the L‐MIND study according to which the majority of AEs are related to lenalidomide. The main extra‐hematological adverse reactions reported in the pivotal study were skin rash in 29 patients and diarrhea in 27 patients, while among the patients in the TALOs study there were 4 cases of skin rash and 4 cases of diarrhea [12]. However, AE incidence were reported in different manner, that is rate were calculated on patients in the L‐MIND study (particularly in at least 10% of patients) whereas in TALOs on the total of AEs themselves reporting how many patients had toxicities [12].

This issue is just one of those which preclude robust comparisons between studies, especially in real‐world reports. Another issue, for example, is the median follow‐up, reported in some cases as pure median, in other cases only for patients still alive or estimated with the reverse Kaplan Meier method as in our case.

Nevertheless, even if they are preliminary results and despite small sample size, data may help to contextualize emerging therapies with a desirable effective flowchart for R/R DLBCL [6, 23].

The results of the TALOs study are similar to those of other (few) retrospective studies (mainly congress abstracts) on the combination of tafasitamab and lenalidomide carried out in other countries and presented over the last few years, also conducted on patients with higher risk characteristics compared to the study sample L‐MIND and therefore with inferior results compared to the pivotal study.

The study by Qualls et al. was conducted on 178 patients with R/R LBCL with a median age of 75 years [16]. Among these there were 68 patients (38.2%) previously undergoing at least 3 lines of therapy, 52 patients (29.2%) undergoing prior CAR‐T therapy and 21 patients (11.8%) with ECOG performance status > 2. Other characteristics of the sample were the IPI score ≥ 3 in 64% of patients and the advanced stage of the disease in the majority of cases (stage III ‐ IV in 83.7% of cases); 87 patients (48.9%) were refractory to the first line of therapy and 118 patients (66.3%) were refractory to the last therapy. In this study population, final OR and CR rates of 31% and 19% respectively emerged, in line with the results of the TALOs study (32.6% and 20.5%). The median PFS was 1.9 months and the median OS was 6.5 months with a median follow‐up of 12 months. Similar to the TALOs study, also in this case the toxicity rates do not differ significantly from those reported in the L‐MIND study despite the higher risk characteristics and major associated comorbidities. This could be due to the initial reduction of the dose of lenalidomide (in 63% of cases), to the early interruption of therapy due to disease progression or to the underestimation due to the retrospective collection of data relating to toxicity [16].

The study by Ruckdeschel et al. included 127 patients with R/R DLBCL, with a median age of 73 years [18]. Many of these patients (70 patients [55.1%]) had undergone at least 3 previous lines of therapy and some (24 patients) also underwent CAR‐T cell therapy. Furthermore, 94 patients (74%) had elevated LDH values, 70 patients (55.1%) had an IPI score ≥ 3 at diagnosis and 55 patients (43.3%) were refractory to the first therapy. This study showed an ORR of 33.1% and a CRR of 11.8%, a median PFS of 4.7 months and a median OS of 8.9 months with a median follow‐up of 10.1 months. Also in this case the data relating to final response rates and survivals are in line with the results emerging from the TALOs study. Regarding toxicities, the results are comparable to those of the L‐MIND study: on a subsample of 118 patients, 74 cases (62.7%) of hematological adverse reactions and 51 cases of extra‐hematological toxicities were observed, in particular 39 patients (33%) developed infections and 12 patients (10.2%) developed skin rash [18].

The study by Saverno et al. and its subsequent update was conducted on a sample of 181 patients with a median age of 71.1 years [14, 15]. Among these, 6 patients (3.3%) had undergone previous treatment with CAR‐T and 86 patients (47.5%) had an ECOG performance status ≥ 2. The majority of patients (136 patients [75.1%]) had an IPI score ≥ 3 and an advanced disease stage according to Ann Arbor at diagnosis (169 patients [93.4%] in stage III ‐ IV); furthermore, 47 patients (26%) had primary refractory disease and 59 patients (32.6%) were refractory to the last previous therapy. The results of this study are an ORR of 75.6% and a CRR of 18%. The median PFS was 11.3 and median OS 24.8 months, respectively with a median follow‐up of 15 months [14, 15]. No further comparisons can be made as they are only congress abstracts.

The GELTALMO group reported a PFS of 10.9 months, better in non‐refractory patients and prolonged survivals in CR patients as in our study [20].

The last real‐world study published was on the French early access program of (2022) [17]. Results were similar to the TALOs study as, at median follow‐up of 8.2 months, best ORR was 46.8%, with CR rate of 29%. Median PFS and OS were 4.7 and 10 months, respectively in the overall population, and long‐lasting responses were observed in patients achieving CR.

The TALOs study has limitations related to its retrospective and multicenter nature, such as the heterogeneity in patient selection and the lack of standardization of clinical management, as the patients were treated in different Centers. Although to be confirmed with further studies with a larger study sample, those of the TALOs study are important results as they confirmed how the use of this therapy can represent a valid option in the treatment of patients with R/R DLBCL, especially with low‐risk disease characteristics and few comorbidities. In fact, comparing the data of the L‐MIND registration study with those of the TALOs study and other retrospective studies conducted in a real‐world context, it emerges from our detailed analyses that the main differences are linked to the characteristics of the disease and the general conditions of the enrolled patients, which they can therefore also justify the differences in terms of outcome. Another interesting message ‐ not previously reported ‐ is that, when toxicities occur, it seems a more appropriate choice to continue treatment at a reduced dose rather than discontinue tafa‐lena. This was shown by a positive relation between dose reduction with DoR, PFS and OS, in contrast with a very short median TTNT for patients who discontinued treatment.

## Author Contributions

L.A. and P.L.Z. conceived the study. C.P., O.A., P.A., E.A., F.P., F.B., A.B., R.B., C.C., G.C., G.D., E.D., A.F., F.G., V.G., A.G., L.E.V., E.L., F.M., L.N., P.N., M.N., F.P., C.P., V.O., M.R., F.R., G.S., A.S., M.T., D.V., B.C., and A.B. followed study patients. L.A., C.P. and P.L.Z. collected data and interpreted the results: L.A. performed statistical analysis: L.A. and C.P. wrote the manuscript: L.A. and P.L.Z. supervised the project: B.C., A.Bro. and P.L.Z. revised the manuscript. All authors approved the final manuscript.

## Funding

The work reported in this publication was funded by the Italian Ministry of Health, RC‐2025–2797392 project.

## Ethics Statement

The start of patient recruitment/selection was subjected to and followed the approval of the study by the Ethics Committee of the Coordinating Center (approval id 442/2023/Oss/AOUBo). This study adhered to the standards of the Helsinki Declaration.

## Consent

All patients were enrolled consecutively after providing written informed consent or authorization from the privacy guarantor for patients who were lost to follow‐up or died.

## Conflicts of Interest

The authors declare no conflicts of interest.

## Supporting information


Supplementary Information S1

## Data Availability

The data that support the findings of this study are openly available in Zenodo at https://zenodo.org/records/15878820, reference number 15878820.
