# Supplementary material for: TALOs, Fill the Gap: Tafasitamab and Lenalidomide in Diffuse Large B‐Cell Lymphoma in the Real‐Life Patient Journey
Source: Hematol Oncol. 2026 Jan 14;44(1):e70167. doi: 10.1002/hon.70167 (PMC12801173; doi:10.1002/hon.70167)
Supplement: Supplementary file 1 — Supplementary Information S1 [file HON-44-e70167-s001.docx]

**Supplementary Results**

In the Cox stepwise backward regression, for OS, the only statistically significant predictor is ECOG (2-3), with an HR of 2.529; patients with an ECOG level of 2-3 have a death risk 2.5 times higher than patients with a level of 0-1 (Table S1). In the case of PFS, the variables Bulky Disease and Double-hit are significant. Patients who exhibited Bulky Disease had a risk of disease progression or death that was 3.5 times greater than those who did not present it; similarly, the risk of disease progression or death in patients with Double-hit was found to be 4.4 times higher (Table S2).

**Supplementary Tables**

**Table S1**. Cox stepwise backward model for Overall Survival

|  | Multivariate | |
| --- | --- | --- |
|  | HR | *p* |
| Sex (male) | 1.975 | 0.055 |
| Age (>=70)  At diagnosis  At baseline | 1.067  1.478 | 0.884  0.431 |
| Primary refractory | 1.685 | 0.096 |
| ECOG PS (2-3) | 2.529 | **0.012** |

ECOG PS: Eastern Cooperative Oncology Group performance status; HR: hazard ratio.

**Table S2**. Cox stepwise backward model for progression-free survival

|  | Multivariate | |
| --- | --- | --- |
|  | HR | *p* |
| Sex (male) | 0.861 | 0.637 |
| Age (>=70 years)  At diagnosis  At baseline | 0.891  1.191 | 0.763  0.691 |
| Primary refractory | 2.069 | 0.014 |
| Bulky disease | 3.576 | **0.000** |
| Double-hit | 4.414 | **0.001** |

HR: hazard ratio.
